# Assessing the Performance of GPT-3.5 and GPT-4 on the 2023 Japanese Nursing Examination

**DOI:** 10.7759/cureus.42924

**Published:** 2023-08-03

**Authors:** Yudai Kaneda, Ryo Takahashi, Uiri Kaneda, Shiori Akashima, Haruna Okita, Sadaya Misaki, Akimi Yamashiro, Akihiko Ozaki, Tetsuya Tanimoto

**Affiliations:** 1 College of Medicine, Hokkaido University, Hokkaido, JPN; 2 Department of Rehabilitation Medicine, Sonodakai Joint Replacement Center Hospital, Tokyo, JPN; 3 Department of Foreign Languages, Dokkyo University, Saitama, JPN; 4 Department of Obstetrics and Gynecology, Shonan Kamakura General Hospital, Kanagawa, JPN; 5 College of Medicine, Tokyo Women’s Medical University, Tokyo, JPN; 6 Department of Rehabilitation Medicine, Sonoda Daiichi Hospital, Tokyo, JPN; 7 Department of Nutrition Science, Shokei Gakuin University, Miyagi, JPN; 8 Department of Breast and Thyroid Surgery, Jyoban Hospital of Tokiwa Foundation, Fukushima, JPN; 9 Department of Internal Medicine, Navitas Clinic, Kanagawa, JPN

**Keywords:** chatgpt-4, chatgpt-3.5, gpt-3.5, gpt-4, japan, clinical applications, national nursing examination, ai & robotics healthcare, chatgpt

## Abstract

Purpose

The purpose of this study was to evaluate the changes in capabilities between the Generative Pre-trained Transformer (GPT)-3.5 and GPT-4 versions of the large-scale language model ChatGPT within a Japanese medical context.

Methods

The study involved ChatGPT versions 3.5 and 4 responding to questions from the 112th Japanese National Nursing Examination (JNNE). The study comprised three analyses: correct answer rate and score rate calculations, comparisons between GPT-3.5 and GPT-4, and comparisons of correct answer rates for conversation questions.

Results

ChatGPT versions 3.5 and 4 responded to 237 out of 238 Japanese questions from the 112th JNNE. While GPT-3.5 achieved an overall accuracy rate of 59.9%, failing to meet the passing standards in compulsory and general/scenario-based questions, scoring 58.0% and 58.3%, respectively, GPT-4 had an accuracy rate of 79.7%, satisfying the passing standards by scoring 90.0% and 77.7%, respectively. For each problem type, GPT-4 showed a higher accuracy rate than GPT-3.5. Specifically, the accuracy rates for compulsory questions improved from 58.0% with GPT-3.5 to 90.0% with GPT-4. For general questions, the rates went from 64.6% with GPT-3.5 to 75.6% with GPT-4. In scenario-based questions, the accuracy rates improved substantially from 51.7% with GPT-3.5 to 80.0% with GPT-4. For conversation questions, GPT-3.5 had an accuracy rate of 73.3% and GPT-4 had an accuracy rate of 93.3%.

Conclusions

The GPT-4 version of ChatGPT displayed performance sufficient to pass the JNNE, significantly improving from GPT-3.5. This suggests specialized medical training could make such models beneficial in Japanese clinical settings, aiding decision-making. However, user awareness and training are crucial, given potential inaccuracies in ChatGPT's responses. Hence, responsible usage with an understanding of its capabilities and limitations is vital to best support healthcare professionals and patients.

## Introduction

Artificial intelligence (AI) represents rapid growth, transforming many aspects of our lives, including the medical field. Utilizing advanced algorithms and machine learning, AI potentially aids physicians in making more accurate diagnoses, identifying potential health risks, and providing patients with personalized treatment plans [1-5]. Notably, Chat Generative Pre-trained Transformer (ChatGPT)-3.5 [6], a large-scale language model (LLM) developed by OpenAI and launched on November 30, 2022, is the first of its kind readily accessible to the general public, and its potential applications in healthcare and medical scenarios are currently under investigation. Specific areas of interest include healthcare and health management documentation, data interoperability, diagnostics, research, and education [7]. Measuring such capabilities involves solving test problems and evaluating performance [8, 9].

Moreover, OpenAI released GPT-4, the latest version of the large-scale language model that powers ChatGPT, on March 14, 2023. Internal evaluations reported that GPT-4 saw an 82% decrease in the likelihood of responding to unauthorized content requests and a 40% increase in the probability of producing fact-based answers compared to GPT-3.5 [10]. One significant upgrade is the ability to handle not only text but also images. It was reported to have passed the United States Bar legal exam with results in the ninetieth centile compared to the tenth centile for the previous version of ChatGPT [10]. However, studies comparing the performance changes between GPT-3.5 and GPT-4 in Japanese medical settings are limited.

In order to examine the changes in capabilities between GPT-3.5 and GPT-4 in the context of a Japanese-speaking medical setting, we used the 112th Japanese National Nursing Examination (JNNE) [11], which is a paper-based theory examination conducted in February 2023, as a basis for our evaluation. This examination, conducted following the Act on Public Health Nurses, Midwives, and Nurses [12], is overseen by the Japanese Ministry of Health, Labor, and Welfare (MHLW). The test criteria aim to concretely demonstrate the essential knowledge that nurses should at least share when they first step into the nursing field, ensuring the minimum level of competency that nurses in Japan should possess in the theoretical aspect [13]. Accordingly, this study aimed to evaluate the usefulness of ChatGPT in the Japanese clinical environment by assessing the difference in capabilities between GPT-3.5 and GPT-4 and testing the accuracy rate in response to questions from the JNNE.

## Materials and methods

ChatGPT

ChatGPT (OpenAI LLC, San Francisco, CA, USA) is an artificial intelligence language model that became widely available in November 2022. It is an AI that generates instant, natural conversation-style responses to queries [6]. The number of users is estimated to have exceeded 100 million as of June 2023. In response to input, it learns and analyzes vast amounts of language data from various sources and can generate outputs that are human-like, with the quality of its responses being evaluated. The utilization of the GPT-3.5 version of the installed large language model (LLM) is free, but as of July 2023, users can opt for the GPT-4 version by paying a monthly fee of 20 USD.

Japanese National Nursing Examination (JNNE)

The JNNE has a total examination time of five hours and 20 minutes, divided into morning and afternoon sessions with 120 questions each, totaling 240. Eleven subjects are covered in the examination, including the structure and function of the human body, understanding disease and promoting recovery, health support and social security systems, basic nursing, adult nursing, geriatric nursing, pediatric nursing, maternal nursing, psychiatric nursing, home care nursing theory, and integrated and practical nursing.

The examination comprises 50 compulsory questions that probe fundamental medical knowledge, 130 general questions in a question-and-answer format covering all 11 subjects established in the test criteria, and 60 scenario-based questions. The scenario-based questions set up scenarios that one could face in a nursing setting for seven subjects (excluding structure and function of the human body, understanding disease and promoting recovery, health support and social security systems, and basic nursing as defined in the examination criteria) and probe the examinee's understanding and judgment in response to these situations.

The basic format of JNNE is multiple-choice with four options, but a few questions with five options (select one or select two) are also presented. A few calculation problems are included, and some conversation questions, which simulate dialogues with patients in the actual clinical setting, are also part of the exam. Questions are deemed 'inappropriate' if they have multiple correct answers, are excessively difficult, or the situation is not adequately set up to obtain a correct answer despite being a multiple-choice question. Depending on the reason for being labeled as inappropriate, measures include excluding all examinees from scoring, only including correct responses for scoring (incorrect responses are excluded), or treating multiple options as the correct answer. According to the official announcement, one question was excluded from scoring in the 112th JNNE, and another question was considered to have multiple answers [13]. Therefore, we excluded these two questions from the analysis in order to evaluate ChatGPT's performance more precisely.

Compulsory and general questions are scored one point per question, while scenario-based questions are scored two points. The passing criteria are set as answering correctly to over 80% of compulsory questions and meeting the annual standard for the total score of general or scenario-based questions. Specifically, about 65% of the points are required for the latter. In the 112th National Nursing Examination, which we used for our analysis and was conducted in February 2023, the passing criteria were 40 points or more for compulsory questions and a total score of 152 points or more for general and scenario-based questions. The exam had 64,051 candidates, 58,152 of whom passed, making the pass rate 90.8% [13].

Analysis

The test questions from the 112th JNNE [11], administered on February 12, 2023, were allowed to be taken home by the authorities, and the examinees provided actual questions for research purposes. The questions were manually inputted into the interface of ChatGPT, and responses were subsequently generated utilizing both the GPT-3.5 and GPT-4 iterations of ChatGPT on the date of March 31, 2023. It has also been noted that ChatGPT learns from context and that the type of answer obtained from a previous question may influence the next question. In order to reduce such influence, all questions were filled out on a new form, with the application updated each time an answer was given, and ChatGPT outputs the answer. In addition, as scenario-based questions are posed in sets of three, ChatGPT was asked to answer them all together by inputting the first question, followed by the second and third. Furthermore, for questions that included images, in order to compare the specs with GPT-3.5, only the question text was entered without using image information. The correctness of the answers produced by ChatGPT was determined based on official announcements from the MHLW [11]. In this study, three analyses were performed. Firstly, the correct answer rate and score rate were calculated for each question type as well as for the entire examination. Next, the correct answer rate for each question was compared for the GPT-3.5 and GPT-4 cases. Finally, the correct answer rates for conversation questions were compared when solved by GPT-3.5 and GPT-4, respectively. Based on the previous study, the McNemar test was utilized to conduct comparisons between the rates of correct responses [14]. All tests were two-tailed, and statistical significance was determined at a p-value less than 0.05. Stata version 15.0 (StataCorp LLC, College Station, TX, USA) was used for all data analyses.

Ethical approval

This study solely utilized data previously published online and did not involve any human subjects. Instead, an analysis of the JNNE was conducted. Therefore, ethical considerations were not applied to this study.

## Results

Out of 238 questions posed in Japanese, both versions of ChatGPT, GPT-3.5, and GPT-4 were able to generate some form of answers for the same 237 questions. The question for which an answer could not be generated came from a general question that required image information to be answered and was excluded from the analysis. Thus, the assessment was carried out with a total of 237 questions. Among them, one question was a mathematical problem, and 15 were conversation questions.

The overall accuracy rate was 59.9% (142/237) for GPT-3.5 and 79.7% (189/237) for GPT-4, showing a statistically significant difference (p<0.01). In terms of scoring, for both compulsory and general/scenario-based questions, GPT-3.5 achieved 58.0% (29/50) and 58.3% (144/247), respectively, failing to meet the passing standard in both categories. In contrast, GPT-4 scored 90.0% (45/50) and 77.7% (192/247), respectively, satisfying the passing standard in both categories.

Table 1 shows the accuracy rates for each problem type.

**Table 1:** The percentage of correct answers for questions in each category GPT: Generative Pre-trained Transformer

	GPT-3.5	GPT-4	p-value
Compulsory Questions	58.0% (29/50)	90.0% (45/50)	p<0.01
General Questions	64.6％ (82/127)	75.6% (96/127)	p=0.014
Scenario-Based Questions	51.7% (31/60)	80.0% (48/60)	p<0.01
Total Accuracy Rate	59.9% (142/237)	79.7% (189/237)	p<0.01

For compulsory questions, the accuracy rates were 58.0% (29/50) for GPT-3.5 and 90.0% (45/50) for GPT-4 (p<0.01).

For general questions, they were 64.6% (82/127) for GPT-3.5 and 75.6% (96/127) for GPT-4 (p=0.014). For scenario-based questions, they were 51.7% (31/60) for GPT-3.5 and 80.0% (48/60) for GPT-4 (p<0.01). In all categories, GPT-4 demonstrated a higher accuracy rate than GPT-3.5.

Table 2 shows the matches between correct and incorrect GPT-3.5 and GPT-4 answers for questions in each category.

**Table 2:** The matches between correct and incorrect GPT-3.5 and GPT-4 answers for questions in each category GPT: Generative Pre-trained Transformer

	GPT-3.5 Correct (%)	GPT-3.5 Incorrect (%)
Compulsory Questions		
GPT-4 Correct	28 (56.0%)	17 (34.0%)
GPT-4 Incorrect	1 (2.0%)	4 (8.0%)
General Questions		
GPT-4 Correct	75 (59.1%)	21 (16.5%)
GPT-4 Incorrect	7 (5.5%)	24 (18.9%)
Scenario-Based Questions		
GPT-4 Correct	28 (46.7%)	20 (33.3%)
GPT-4 Incorrect	3 (5.0%)	9 (15.0%)
Total		
GPT-4 Correct	131 (55.3%)	58 (24.5%)
GPT-4 Incorrect	11 (4.6%)	37 (15.6%)

Both GPT-3.5 and GPT-4 successfully generated correct answers for a total of 131 questions (46.4%). Additionally, there were 58 questions (24.5%) where GPT-3.5 gave incorrect answers that GPT-4 provided correct answers for, and there were 37 questions (15.6%) where both versions provided incorrect answers. On the other hand, there were 11 (4.6%) questions where GPT-3.5 provided correct answers while GPT-4 returned incorrect ones. In the category of compulsory questions, there were 17 instances (34.0%); in general questions, 21 instances (16.5%); and in scenario-based questions, 20 instances (33.3%), where questions that were incorrectly answered by GPT-3.5 were correctly answered by GPT-4.

In terms of conversation questions, the accuracy rate was 73.3% (11/15) for GPT-3.5 and 93.3% (14/15) for GPT-4 (p=0.248). Neither achieved a correct answer to the mathematical problem. The mathematical problem was not exported with the correct answer in both versions.

## Discussion

In this study, we assessed the accuracy of ChatGPT versions GPT-3.5 and GPT-4 using the multiple-choice format of the JNNE administered in February 2023. Although the accuracy of randomly chosen answers for this exam stands at approximately 20%-25%, the overall accuracies for GPT-3.5 and GPT-4 were recorded at 59.9% (142/237) and 80.2% (190/237), respectively, both significantly exceeding the likelihood of random selection. While GPT-3.5 did not meet the passing requirements of the JNNE, GPT-4 exceeded the passing criteria. This suggests the potential utility of the latter in assisting with clinical diagnoses and treatment decisions in real-world Japanese medical settings, provided its use is approached with caution and an understanding of its inherent characteristics.

The documented 80% accuracy rate in scenario-based questions, in conjunction with a 93.3% accuracy rate in conversation questions, bolsters the proposition that ChatGPT could potentially augment medical professionals in delivering personalized treatment plans and facilitating remote patient care [15, 16]. Moreover, given the challenging working conditions for medical professionals, exacerbated by burnout and workforce shortages amid the COVID-19 pandemic [17], such AI systems could contribute to ameliorating the working environment. However, at the same time, it should be noted that the accuracy of ChatGPT is not fully guaranteed, and there have been reports of it potentially providing erroneous information in a naturalistic manner, a phenomenon known as 'hallucination' [18, 19]. In addition, it has been reported that there is room for improvement in the performance concerning more specialized domains [20]. Hence, uncritically accepting all information generated by the system would be risky, and from this perspective, it is deemed necessary at the current stage not to consider its deployment in the medical field immediately but to persistently conduct specialized training, thereby enhancing its performance in the specific medical field. Furthermore, its wide accessibility to patients raises the potential risk of disseminating incorrect health information, necessitating due caution.

The accuracy of ChatGPT has been reported to improve with a certain degree of training [21]. Indeed, an approximate 20% increase in accuracy was noted following the introduction of GPT-4, a mere half a year after the debut of GPT-3.5. As the user base grows and specialized training accumulates, ChatGPT could become a valuable tool in Japanese clinical settings. However, healthcare professionals' functions, such as information evaluation and patient communication, will remain essential responsibilities regardless of AI advancements [22]. Therefore, proactive engagement from healthcare professionals to utilize reinforcement learning with AI in actual clinical settings is indispensable for leveraging ChatGPT as a more reliable source of medical information.

Additionally, while direct comparisons are challenging, it is worth noting that GPT-4 recorded an 80.2% accuracy rate on the JNNE in this study, while its accuracy rate on the United States Medical Licensing Examination Educational (USMLE) entrance examination was reported to exceed 90% [7]. This implies that the performance of ChatGPT may be influenced by the language through which information is conveyed. Indeed, it is estimated that the amount of information accessible in English on the Internet is approximately 16.6 times greater than in Japanese [23]. Due to these factors, the varying treatment strategies influenced by different cultural backgrounds may not be sufficiently reflected in the responses generated by ChatGPT. The approaches to health can vary by culture, with some cultures prioritizing a holistic or non-invasive approach to treatment while others are more tolerant of surgical interventions [24]. However, caution should be paid because if the volume of linguistic information sways the content generated by ChatGPT, it might not adequately respond in line with such cultural contexts without careful consideration, such as the proper use of prompts [25].

Further, the discrepancy in accuracy rates might be attributable to cultural nuances inherent in the Japanese examination system. The Japanese medical examination may require significant degrees of implicit understanding from the examiners to attain high scores, given that the exam may not always explicitly indicate correct or incorrect responses. Japan, recognized as a high-context communication society, often involves interpreting the atmosphere and anticipating unexpressed intentions due to shared cultural norms [26]. This cultural context may influence the formulation of exam questions, necessitating examinees to infer implicit knowledge that is not explicitly stated.

This study has several limitations. First, we did not scrutinize the basis of the answers in this analysis. Since most of the exam questions are multiple-choice, there is a possibility that the correct answer could be reached by chance. In the future, it will be important to verify the validity of the basis of ChatGPT's correct answers and reveal its performance in more detail. Second, in this study, the trial was conducted only once, based on other studies conducted in a similar manner, and the results were analyzed. However, ChatGPT may output different answers each time a question is asked due to the language model behind the main architecture. Therefore, in order to reduce errors caused by this, it may be possible to measure ChatGPT performance more rigorously by attempting the JNNE several times and comparing the percentage of correct answer rates in each of these attempts. Third, a detailed evaluation of questions based on correct or incorrect responses, or a detailed analysis by field, was not performed. Investigating the characteristics of problems where GPT-3.5 was incorrect and GPT-4 was correct, as well as differences in accuracy rates by domain, through trials based on larger datasets, could potentially reveal areas in need of further enhancement. Therefore, it is important to focus on these aspects as areas of consideration for future studies. Fourth, it is important to acknowledge that ChatGPT versions are constantly evolving. With subsequent versions beyond GPT-3.5 and GPT-4, modifications to the LLMs could potentially lead to significant changes in the time required for generating responses and their quality [27]. Despite these limitations, we believe that the study was able to be conducted at an adequate level, with doctors, nurses, physiotherapists, etc. at the center of the research.

## Conclusions

Our study demonstrated that while the GPT-3.5 version of ChatGPT did not meet the criteria, the GPT-4 version already exhibits the performance required to pass the JNNE. The fact that the accuracy rate dramatically improved in less than half a year suggests that by executing training specialized in the medical field in Japanese, such large language models could potentially become beneficial sources of information in clinical decision-making in Japanese clinical settings, assisting medical professionals in diagnosing and determining treatment plans, and helping patients decide whether to visit a hospital or not. However, given that accuracy is not assured and the hallucinations generated by ChatGPT can be convincingly naturalistic, there is a potential for erroneous judgments to be made unless the messages are meticulously examined by an experienced healthcare professional. Therefore, when implementing in a clinical environment, not only the training of the language model but also the training and awareness-raising of the users will be important. It is crucial to ensure that these tools are used responsibly and with a clear understanding of their capabilities and limitations to provide the best support to healthcare professionals and patients alike.
